# Septic encephalopathy: when cytokines interact with acetylcholine in the brain

**DOI:** 10.1186/2054-9369-1-20

**Published:** 2014-09-01

**Authors:** Qing-Hong Zhang, Zhi-Yong Sheng, Yong-Ming Yao

**Affiliations:** Department of Microbiology and Immunology, Burns Institute, First Hospital Affiliated to the Chinese PLA General Hospital, Beijing, 100048 P.R. China

**Keywords:** Septic encephalopathy, Acetylcholine, Neuroinflammation, Cholinergic anti-inflammatory pathway, Delirium, Immunosuppression

## Abstract

Sepsis-associated encephalopathy (SAE) is a brain dysfunction that occurs secondary to infection in the body, characterized by alteration of consciousness, ranging from delirium to coma, seizure or focal neurological signs. SAE involves a number of mechanisms, including neuroinflammation, in which the interaction between cytokines and acetylcholine results in neuronal loss and alterations in cholinergic signaling. Moreover, the interaction also occurs in the periphery, accelerating a type of immunosuppressive state. Although its diagnosis is not specific in biochemistry and imaging tests, it could potentiate severe outcomes, including increased mortality, cognitive decline, progressive immunosuppression, cholinergic anti-inflammatory deficiency, and even metabolic and hydroelectrolyte imbalance. Therefore, the bilateral communication between SAE and the multiple peripheral organs and especially the immune system should be emphasized in sepsis management.

## Introduction

A previously healthy young woman was transferred to our emergency department 21 days after a third degree thermal injury involving 35% total body surface area. She had been well until one week later after the injury, when a high fever, rapid shallow breathing, excessive anxiety, and hallucinations occurred. She was found to be drowsy and confused when roused, with cold and clammy chin and cyanosis. Her temperature was 38.9°C, with blood pressure of 67/45 mm Hg, and her white blood cell count was 14000 per cubic millimeter. After admission, wound sepsis arising from *P. Aeruginosa* infection was confirmed. Since infection can cause similar neuropsychiatric complications as drugs with anticholinergic effects [1], the mental condition of this patient prompted us to undertake an extensive review of the literature concerning the relationship between infection and acetylcholine in sepsis induced mental conditions.

With advances in modern intensive care that provide vital-organ support and better surveillance, especially the prompt initiation of therapy for the prominent infection, mortality from severe sepsis and septic shock have been reduced to 20% ~ 30% in many reports [2]. With a lowering of mortality rate, attention has now been focused on the quality of life among survivors. Numerous studies have demonstrated that patients who survive sepsis have an increased risk for poor outcomes in the following months and years, with significantly pronounced physical and neurocognitive decline, mental disorders, and long-term morbidity low quality of life [3]. Abnormalities in cognitive domains of executive function, attention, and memory appear to be the most common disorders, and have been demonstrated to occur as long as 6 years after discharge from hospital [4].

Increasing evidence indicates that the brain is not merely a privileged organ in a reversible septic illness, but is one of the organs profoundly affected in an early and progressive manner. Sepsis-associated encephalopathy (SAE) is a brain dysfunction that occurs secondary to infection in the body without overt central nervous system (CNS) infection. The severity of SAE may range from mild delirium to deep coma, and is characterized by changes in behavior, cognition, awareness, and consciousness. Therefore, the term sepsis-associated delirium has recently been proposed to replace the term septic encephalopathy [1]. Sepsis-associated delirium is one of the most common causes of delirium in intensive care units (ICU) [5], and SAE is associated with increased mortality, morbidity and plausibly with diminished long-term cognitive performance [6].

To date, the complex cascade of molecular and cellular events, including those leading to septic encephalopathy after burn injury remain obscure. Furthermore, no effective therapy has been developed. This review is aiming to discuss sepsis-induced brain damage and its recent perception of its pathogenesis, diagnosis, prognosis, sequelae and possibly effective prevention in critical ill patients.

### Epidemiology

Sepsis is one of the leading causes of ICU admissions and SAE is commonly seen in the ICU [7]. As sedation and other treatments often obscure its neurological picture, it might be difficult to recognize delirium in patients with sepsis. Therefore, its reported incidence varies considerably, ranging from 8% to 70%, and the variation is at least in part due to differences in diagnostic criteria.

A recent international survey showed that the prevalence of delirium was 32.3% in the ICU [8]. The incidence of encephalopathy is higher in patients who have bacteraemia and evidence of renal, hepatic or multiorgan failure. 70% of patients with bacteraemia have neurological symptoms ranging from lethargy to coma, and >80% of them showed electroencephalography abnormalities. Patients in the medical and surgical ICUs are at high risk for long-term cognitive impairment. In a retrospective study from China [9], the incidence of SAE was 17.7% in patients admitted to the ICU over a 3-year period. Acute physiology and chronic health evaluation II score, heart rate, blood lactate, serum sodium were significantly higher in the SAE group than those in non-SAE group. While GCS scores, platelet counts, serum albumin level, and pH value were significantly lower in the SAE group [9]. Recently, it was reported that patients with acute lung injury presented impairment of memory, verbal fluency, executive function, and cognition in 13%, 16%, 49%, and 55% respectively, of long-term survivors as assessed for neuropsychological functions at 2 and 12 months after being discharged from hospital. Depression, post-traumatic stress disorder, and anxiety were found to be present in 36%, 39%, and 62% of long-term survivors [10]. 6% of patients in the ICU with respiratory failure or shock showed cognitive impairment, and delirium developed in 74% of them during the hospital stay. Three months after being discharged, 40% of the patients had cognition scores similar to that of patients with moderate traumatic brain injury, and 26% had similar scores as that of patients with mild Alzheimer's disease [5]. Among a cohort of non-demented elder adults, hospitalization due to acute care for critical illnesses also showed greater cognitive decline when compared to that before hospitalization [11].

Burn patients are at high risk of infection, and it is not surprising that burn injuries are associated with a high rate of anxiety, depression, and posttraumatic stress disorder [12]. Disorientation regarding time and place has often been observed at the early period of burn-associated sepsis and about two-thirds of burn survivors exhibit a history of lifetime psychiatric disorders. In a multinomial regression analysis, the percentage of total body surface area injured was shown to be an independently and strongly predicted risk factor for the occurrence of mental disorders, especially anxiety disorders and delirium [13]. Burn injury can also induce neural lesions with microabscess, the release of pro-inflammatory cytokines from glia cells, the cleavage of structural proteins such as gelsolin [14], and even apoptosis in the brain [15]. These events provide the pathological basis for the neuropsychiatric alterations induced by acute burn insults.

### Pathophysiology

#### Neuroinflammatory processes

Development of SAE probably involves a number of mechanisms, including hypoxemia, hypotension, glucose dysregulation, systemic inflammation, reduced cerebral blood flow, disruption of the blood–brain-barrier, (BBB), cerebral edema, abnormal neurotransmitter composition, impaired astrocyte function, and neuronal degeneration. Neuroinflammation, expressed as frank microglial activation with excessive expression of immune cytokines based on brain-immune interactions, is involved in the development of encephalopathy [16, 17]. Systemic inflammation can produce mild cognitive changes in healthy individuals [18] and in severe cases (i.e., sepsis/critical illness) it can trigger delirium [19]. Preliminary studies with brain tissue obtained from 9 patients with delirium was compared to 6 age-matched controls without delirium, and it was found that there was an association of human brain activity of microglia, astrocytes, and IL-6 with delirium in elderly patients. These results provided evidence that inflammatory mechanisms to be involved in delirium [20]. SAE may be a consequence of the activity of inflammatory mediators on neural cells. For instance, peripheral administration of lipopolysaccharide (LPS) to mice was found to cause a rapid and steep rise in TNF-α in the brain that remained elevated for 10 months [21]. As sepsis in patients was associated with elevated levels of TNF-α and interleukins (IL)-1, as well as IL-6 in cerebrospinal fluid, the pathophysiological mechanisms of SAE may be the result of a cascade of neuroinflammatory processes as followed.

The first event is an inflammatory process that starts in the cerebral endothelial lining, which directly releases or, through alteration of the BBB, facilitates the passage of inflammatory mediators into the parenchyma. The exclusively peripheral cytokine signal can be transmitted to the brain through direct neural pathways (*via* primary autonomic afferents), or across the BBB, or *via* the circumventricular region, where the BBB is non-existent or discontinuous [22]. Secondly, immediately upon entering the cerebral parenchyma, the mediators alter cellular metabolism by inducing oxidative stress, mitochondrial dysfunction and microglia activation. This then triggers neuropathologic abnormalities that range from alterations in neurotransmission to apoptosis, and finally the consequent delirium. Moreover, these mediators modulate β-adrenergic, GABAergic, or cholinergic neurotransmission and secretion of corticotropin-releasing factor, adrenocorticotropic hormone, and vasopressin [23], affecting the neuroendocrine pathways and leading to profound systemic response, which in turn aggravate SAE.

There are now multiple studies in clinical settings which showed associations between pro-inflammatory cytokines and delirium, and that a severe systemic inflammation may more likely produce delirium [24–27]. Prior cognitive impairment is found to be the major risk factor for delirium, and such impairment may also be associated with higher levels of pro-inflammatory cytokines [24]. Another study found that delirium after surgery was the result of a dysfunctional neuroinflammatory response [26]. It may partly be a consequence of decreased levels of anti-inflammatory mediators rather than exclusively an excess of pro-inflammatory cytokines [27]. The mechanism by which the inflammatory mediators produce SAE may be through interacting with the important neurotransmitter, acetylcholine (ACh).

#### Cholinergic-dependent septic encephalopathy: Interaction between cytokines and acetylcholine in the brain

Neurotransmission mediated by Ach, in particular, contributes to numerous physiologic functions including memory, learning, and panic responses. Increasing evidence indicates that an interaction between cytokines and acetylcholine is involved in the development of delirium. Experimental findings show that chronic low dose LPS infusion in rats results in extensive neuroinflammation and a substantial reduction in cortical choline acetyltransferase activity, which is a marker of cholinergic integrity. In particular, IL-1 inhibits ACh release in vivo in the hippocampus [28], as well as ACh synthesis in vitro in cultured pituitary cells [29]. Moreover, IL-1 increases acetylcholinesterase activity and mRNA expression in both neuron-glia co-cultures and in the rat cortex *in vivo* [30]. One study demonstrated altered behavior and long-term memory deficits in rats after an injection of bacterial LPS, which may be indicative of reduced cholinergic innervation of the cortex, the hippocampus and the prefrontal cortex [31]. Together, these studies suggest that both neuronal loss and alterations in cholinergic signaling induced by LPS or inflammatory cytokines play potential roles in the long-term effects of SAE.

Recent animal studies also indicated that some delirium might be underpinned by cholinergic hypofunction, perhaps in combination with dopaminergic overactivity [32]. This hypothesis is based on the effectiveness of an anti-cholinergic drug treatment to induce delirium in the clinic. Animal studies further showed that neither animals with neuronal loss in the cholinergic nucleus, nor normal animals challenged with LPS showed any deficits on cognition. However, cholinergic deficient animals, when challenged with LPS, showed acute and transient working memory deficits [33]. These deficits could be partially reversed by the administration of the acetylcholinesterase inhibitor, indicating that systemic inflammation induced impairments via disruption of cholinergic signaling. Thus, prior vulnerability in the cholinergic system made these animals susceptible to cognitive impairments induced by LPS.

Similar to what has been noted in the peripheral tissues, acetylcholine seems to also play a role in control of brain inflammation as microglia express nicotinic receptors and activation of these cholinergic receptors attenuates the pro-inflammatory response [34]. It was not surprising to find that anticholinergic drugs are recognized as risk factors for delirium due to a lack of cholinergic anti-inflammatory effects in the brain [35, 36]. Medications with anticholinergic activity negatively affect the cognitive performance in elderly adults [37], patients with acute stroke, and psychological patients. Many commonly prescribed drugs have anticholinergic effects. Systemic side effects, which are mainly cerebral include visual and tactile hallucinations, incoherent speech, agitation, disorientation, memory loss, and acute psychotic reaction have been described. Based on these observations, it was postulated that systemic inflammatory mediators during sepsis damage cholinergic neurons in the hippocampus and frontal lobe, leading to reduced cholinergic activity on one hand [38], and impaired cholinergic inhibitory control of microglia on the other hand. Together, these events contribute to uncontrolled neuroinflammation leading to delirium and cognitive decline in the long term.

Microglia plays a crucial role in the innate immune response of the brain. The pro-inflammatory cytokines released by microglia exert a vicious cycle of neuroinflammation. On the other hand, activation of the cholinergic receptors on microglia attenuates the pro-inflammatory response in vitro [38]. Therefore, we suppose that the degeneration of cholinergic neurons following SAE impairs cholinergic inhibitory control of microglia, further exacerbating neuroinflammation in SAE.

#### Septic encephalopathy with immunosuppressive status: Interaction between cytokines and acetylcholine in the periphery

**Figure 1:**
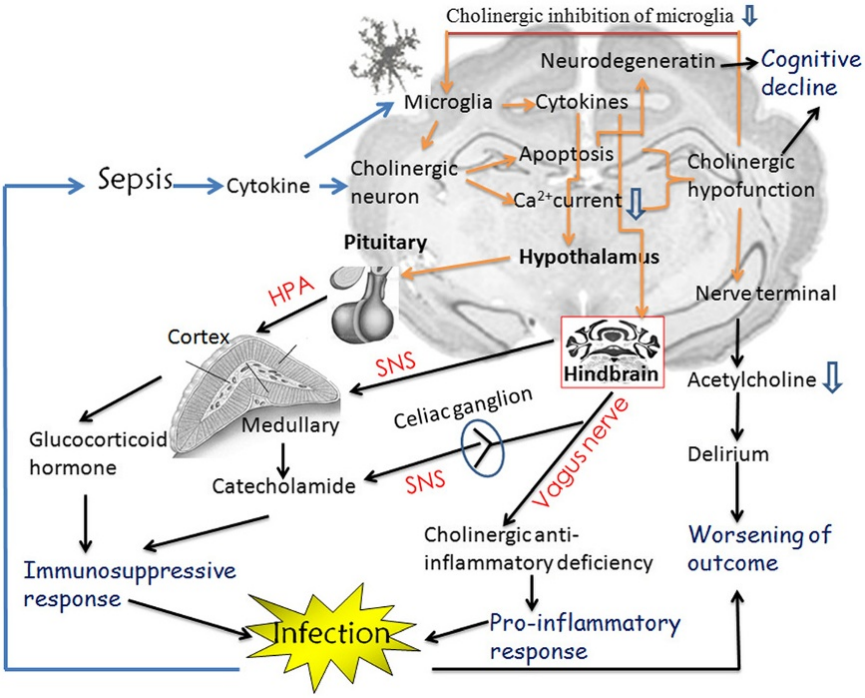
**Interactions of cytokines with acetylcholine in the central nervous system that induces septic encephalopathy and its peripheral consequences.**

The brain senses and modulates the systemic inflammatory response through the neural circuits of vagus nerve via the so-called “inflammatory reflex arc” [22, 39, 40]. The cholinergic neural output has been shown to modulate innate immune response to infection, injury and ischemia through both the release of acetylcholine at the distal vagus nerve acting on macrophages and the connection of the vagus nerve with the spleen *via* the splenic sympathetic nerve (Figure 1). On one hand, vagus nerve stimulation promotes the release of the acetylcholine, which in turn inhibits the systemic inflammatory response by reducing the production of the pro-inflammatory cytokines in human endotoxin-stimulated macrophages [39] *.* On the other hand, recent evidence indicates that the vagus nerve could also activate adrenergic neurons in the spleen. The neurotransmitters (norepinephrine, epinephrine) released by the vagus nerve promote splenic T cells to secrete ACh, which in turn interacts with α7 nicotinic ACh receptors (nAChR) on monocytes and thereby inhibit pro-inflammatory cytokine production [41] *.* Expression of α7 nAChR on other immune cells, including polymorphonuclear neutrophils, dendritic cells (DC), NK cells, B cells, T cells as well as endothelial cells, fibroblasts, and synoviocytes provides a wide range of opportunities for the brain to modulate tissue response to injury [40].

Systemic inflammatory mediators are recruited into the brain *via* the disrupted blood–brain barrier and circumventricular organs, and activate the microglia to produce more cytokines as termed “neuroinflammation”. The consequent cytokines can induce neural apoptosis of cholinergic neuron, which in turn reduces cholinergic activity by decreasing the level of cholinergic neurotransmitter acetylcholine. Cholinergic hypofunction can lead to delirium, cognitive decline, vagus anti-inflammatory deficiency and reduced cholinergic inhibition of activated microglia. The vagus anti-inflammatory deficiency prompts augmented pro-inflammatory response; while the accelerated activated microglia initiates higher levels of cytokines and which in turn activate the hypothalamus-pituitary-adrenal (HPA) axis to release glucocorticoid hormone, and sympathetic nervous system (SNS) to release the catecholamine, both of which lead to immunosuppressive response. The simultaneously occurrence of the enhanced pro-inflammatory and immunosuppressive responses subsequently exacerbate the infection and worsen the outcome.

Accumulating evidence suggested that the “inflammatory reflex arc” might be activated in the situation of encephalopathy. It was recently reported that pathogens can rapidly activate vagal afferent and efferent signaling and cause pronounced bradyarrhythmias [42]. More data suggests that an increase in vagal tone is expected to decrease inflammatory cytokines [43, 44] and catecholamines, reduce ability to clear parasitism [43], as well as lower heart rate variation suppression in a rat sepsis model [44]. Secondly, the intimate interaction between cytokines and acetylcholine in sepsis might reduce the innate immune response during early stages, but if it persists, the consequent enhanced inflammatory reflex would result in immunosuppression. Therefore, immunoparalysis observed in later sepsis may, at least in part, results from enhanced vagal activity and subsequent sustained effects of the cholinergic anti-inflammatory pathway [45]. Additional evidence of activated cholinergic anti-inflammatory pathway in the context of encephalopathy comes from a clinical trial of an acetylcholinesterase inhibitor to treat ICU delirium. The trial was based on the dual role of acetylcholine as a neuromodulator as well as an endogenous inhibitor of systemic inflammatory response to endotoxemia [46]. Unexpectedly, the trial was stopped early because of increased mortality in the treatment group [47]. The possible mechanism for the harmful outcome might due to the accelerated immunoparalysis caused by the enhanced cholinergic anti-inflammatory activity with acetylcholinesterase inhibitor in previous immunosuppressed patients with critical illness. More evidence is critically needed to support this hypothesis.

### Clinical features and diagnosis

The importance of SAE is frequently dismissed, as it is seen as a transient entity. Nevertheless, SAE is often suspected in patients presenting with acutely altered mental status accompanied by severe sepsis or septic shock. The severity of SAE can range from mild delirium to deep coma. They might show disturbances in sleep-wake cycles or evidence of hallucinations, restlessness or agitation, among other symptoms commonly seen in delirium. Moreover, 70% of advanced cases of SAE have an associated critical illness neuromyopathy. In case of burn injury, patients mainly presented with early psycho-motor excitement, characterized by delirium and disorientation, logorrhea, sleeplessness, and loss of self-recognization,

Recently, diffuse and severe white matter abnormalities were present in severe encephalopathy with extensive white matter lesions, though in a reversible condition. Imaging of the brain may show atrophy and periventricular white matter lesions, abnormal low density of the whole white matter with swelling of the entire brain [48]. Thus, SAE remains largely a clinical diagnosis. However, a myriad of abnormalities are seen in patients with SAE, including abnormalities in laboratory tests. A comprehensive metabolic panel of analyses including a complete blood count, measurement of electrolytes and serum enzyme levels, renal function tests, and investigations for infection are required to determine whether the patient has an infection or an anatomical abnormalities [49]. Sepsis-associated delirium was diagnosed using the confusion assessment method in the ICU. For practical purposes, the confusion assessment method for patients outside the ICU was advocated by Young GB [49]. Considering that SAE is an early feature of systemic infection that presents before a satisfactory criteria for sepsis is established, the clinical manifestations of SAE can be detected before presentation of strong evidence of sepsis or systemic inflammatory response syndrome (SIRS).

Although no specific biomarker exists for SAE, several potential biomarkers could be considered. Markers such as neuron-specific enolase (NSE), S100β and glial fibrillary acidic protein (CNS), and their presence in serum reflects CNS injury. Children with sepsis had higher levels of serum NSE, S100 β and GFAP than that of controls, and serum levels of both NSE and S100 β were highest in children who did not survive sepsis [50]. Similar findings have been demonstrated in adults, with 42% of 170 patients with sepsis showing an increase in serum S100 β levels, and 53% of patients showing an increase in serum NSE levels during the first 72 hours after hospital admission [51]. A study showed that increased serum levels of IL-8 were associated with delirium in patients with inflammation, whereas IL-10 and amyloid β levels were elevated in patients with noninflammatory delirium [25]. Patients with inflammatory delirium, who later showed cognitive impairment, had acutely elevated amyloid β levels. Systemic elevations in serum procalcitonin and IL-6 levels were also seen in patients with severe sepsis [52]. However, neither procalcitnonin nor IL-6, is a specific biomarker for CNS injury. Taken together, the above-mentioned studies support the hypothesis that SAE is, in fact, a result of direct CNS injury, and more sensitive biomarker of brain damage is needed.

### Sequelae and outcomes

#### Increased mortality

Meta-analysis has provided evidence that delirium in elderly patients is associated with an increased risk of death, institutionalization, and dementia, independent of age, sex, comorbidity, illness severity, and presence of dementia. Mortality is almost always due to multi-organ failure rather than neurological complications. The persistence of the association between delirium and poor outcome years after the occurrence of delirium and presumably resolution of the precipitating factors suggests that delirium is not merely a marker of underlying disease [6]. In fact, delirium was found to precede the overt diagnosis of sepsis in 30.8% of patients, thus suggesting that delirium might serve as a precursor of the development of sepsis [53], therefore, an increased mortality is expected.

#### Cognitive decline

Mounting evidence in both animal models and in human studies, suggests that substantial long-term cognitive sequelae are associated with SAE. Animal models have shown long-term changes in behavior, learning and memory following SAE [31]. A longer duration of delirium in the hospital was independently associated with long-term cognitive outcomes [54] and worse global cognition as well as executive function scores at 3 and 12 months [5]. Of great importance, longer duration of delirium was recently found to be associated with smaller brain volumes up to 3 months after discharge, and that smaller brain volumes are associated with long-term cognitive impairment up to 12 months [55].

#### Progressive immunosuppression

SAE could lead to progressive immunosuppression possibly by activating sympathetic nerve system, and partially hypothalamic pituitary adrenal (HPA) axis, resulting in uncontrolled infection and vicious cycle with SAE. Additional evidence indicates that sepsis in the later stages can be associated with a state of immunosuppression, broadly defined as lymphopenia and loss of immune function, reflected by the down-regulation of monocytic human leukocyte antigen (HLA)-DR expression, *ex vivo* TNF-α production and elevated plasma IL-10 in parallel [45, 56]. It was clearly revealed that the sympathetic nervous system (SNS) especially β-adrenergic receptors and HPA axis were involved in cerebral trauma-induced immunodepression *via* a mechanism of apoptotic cell death [57]. For instance, experimental stroke induces alterations in the immune system and their outcome *via* and the SNS [58]. Postoperative release of pro-inflammatory cytokines into the cerebrospinal fluid without any signs of systemic inflammation was associated with immunodepression and an increased risk of infections in neurosurgical patients [59]. Direct evidence proved that the pro-inflammatory cytokines such as IL-1β in the brain and cerebral inflammation may trigger a systemic anti-inflammatory condition without preceding systemic inflammation [60]. Persistence of this phenomenon increases the risk for infectious complications. Further clinical and experimental results indicate that pro-inflammatory cytokines produced by damaged brain tissue can directly lead to HPA and SNS activation [61]. Elevated levels of cytokines, such as IL-1β, TNF-α, and IL-6, have been measured after stroke in brain parenchyma and cerebrospinal fluid. Because the autonomic system of the CNS is “hard-wired” with secondary lymphoid organs, interruption of these circuits can result in immune dysfunction. Therefore, multiple causes including the “non-specific” stress response, CNS injury-specific neurogenic signaling, and local CNS inflammation have to be considered as triggers of systemic immunodepression [62].

The neuropathology in SAE is quite similar to that of patients with stroke and traumatic brain injury in terms of elevated levels of pro-inflammatory cytokines in the brain. It prompts us to hypothesize that these cytokines in SAE could also mediate immunosuppression in a similar manner as in brain injury. The irreversible compromised immunity occurred later in severe sepsis could also be the consequence of SNS and HPA activation.

#### Cholinergic anti-inflammatory deficiency

Although the “inflammatory reflex arc” might be activated in the situation of encephalopathy to clear pathogen, SAE in the long term might reduce cholinergic anti-inflammatory activity due to the lack of cholinergic neurotransmission by ACh, resulting in an augmented pro-inflammatory response. Inflammatory reflexes have been proposed as a pathway to facilitate resolution of inflammation triggered by infection, ischemia, or sterile injury and a mechanism of rapid and localized control of immunity [63]. During the early stages of the immune response, the vagus nerve transmits tonic inhibitory activity that attenuates innate responses to injury and the activation of effector cells [64], but when it persists over time, it can lead to immunosuppression which exacerbates SAE. Although clinical studies have not established a causal relationship between depressed vagal nerve signaling and sepsis, lower tonic vagal activity has been associated with decreased capacity to resolve inflammation and with increased morbidity as well as mortality in inflammatory states such as severe sepsis and myocardial infarction [65]. In contrast, inhibition of brain acetylcholinesterase, which causes the increase of Ach could theoretically suppress systemic inflammation through a central muscarinic receptor-mediated and vagal- and α7 nAChR- dependent mechanism [66]. We suppose that at later stages of SAE, the vagus nerve transmitter, acetylcholine, would be decreased due to the neural apoptosis, thus attenuated vagal activity might potentiate a pro-inflammatory response due to the lack of the intrinsic cholinergic anti-inflammatory mechanism.

#### Metabolic and hydroelectrolyte imbalance

Last but not the least, since the CNS could regulate the metabolic and hydroelectric balance, the impairment of neurological function in SAE could also lead to the refractory hyperglycemia [67] and hypernatremia [68] in sepsis.

It is increasingly appreciated that CNS controls the metabolic and electrolyte balance. Reduced CNS insulin signaling from either defective secretion or action, contributes to the pathogenesis of common metabolic disorders, including diabetes and obesity. The hypothalamic IKKβ/nuclear factor kappaB (NF-κB) pathway is a general neural mechanism for the energy imbalance underlying obesity. Forced activation of hypothalamic IKKβ/NF-κB interrupts central insulin/leptin signaling and action, while its suppression significantly protects against obesity and glucose intolerance [69] Hyperglycemia in the ICU has been linked with worse outcomes [70], moreover, NF-κB activity is significantly elevated in adults and children with severe sepsis [71].The impaired insulin signaling and activation of NF-κB prompts us to hypothesize that SAE could also result in metabolic and hydroelectrolyte imbalance.

Disturbances in fluid and electrolytes are among the most common clinical problems encountered in the ICU. Hypernatraemia develops in the ICU because various factors promote renal water loss. For example, relative vasopressin deficiency is seen in approximately one-third of late septic shock patients [68]. Furthermore, hypernatraemia is an independent predictor of mortality [72]. In burn patients, hypernatremia, but not hyponatremia, is an independent predictor of mortality [73]. The possibility for SAE might lead to the disturbance of fluid and electrolytes is that SAE could damage the crucial brain regions regulating the balance of fluid and electrolytes. It was appreciated that dysfunction of the hypothalamus, pituitary, or adrenal glands is a common secondary cause for sodium or water imbalance as a result of trauma, tumors, or inflammation. Moreover, a recent study found that hypernatremia is relevant to the sodium-level-sensing mechanism in the brain, as a patient with autoantibody directed against Nax channel in the brain had essential hypernatremia [74]. Therefore, SAE may disrupt the central sodium-level sensor of body fluids in the brain, making the patients prone to developing hypernatremia.

### Perspectives

Currently, patients with septic encephalopathy receive supportive therapy and therapy for the underlying disease without intervention targeted to the cause of the encephalopathy. Several aspects deserve to be emphasized, in particular the interaction of the development of encephalopathy with the peripheral multiple organ including immune system dysfunction. An improved understanding on the cause of septic encephalopathy would lead to the development of new specific therapy options in the future.

## Abbreviations

SAE: Sepsis-associated encephalopathy
BBB: Blood brain barrier
CNS: Central nervous system
ICU: Intensive care units
LPS: lipopolysaccharide
IL: Interleukins
ACh: Acetylcholine
nAChR: Nicotinic ACh receptors
DC: Dendritic cells
HPA: Hypothalamus-pituitary-adrenal
SNS: Sympathetic nervous system
SIRS: Systemic inflammatory response syndrome
NSE: Neuron-specific enolase
GFAP: Glial fibrillary acidic protein
HLA: Human leukocyte antigen
NF-κB: Nuclear factor kappaB.
